# Role of extracellular matrix architecture and signaling in melanoma therapeutic resistance

**DOI:** 10.3389/fonc.2022.924553

**Published:** 2022-09-02

**Authors:** Ana Popovic, Sophie Tartare-Deckert

**Affiliations:** ^1^ Université Côte d’Azur, Institut National de la Santé et de la Recherche Médicale (INSERM), Centre Méditerranéen de Médecine Moléculaire (C3M), Nice, France; ^2^ Team Microenvironnement, Signaling and Cancer, Equipe Labellisée Ligue Contre le Cancer, Nice, France

**Keywords:** tumor stroma cross-talk, ECM remodeling, ECM mediated therapeutic resistance, melanoma associated fibroblasts, melanoma progression and phenotypic switching

## Abstract

The extracellular matrix (ECM) is critical for maintaining tissue homeostasis therefore its production, assembly and mechanical stiffness are highly regulated in normal tissues. However, in solid tumors, increased stiffness resulting from abnormal ECM structural changes is associated with disease progression, an increased risk of metastasis and poor survival. As a dynamic and key component of the tumor microenvironment, the ECM is becoming increasingly recognized as an important feature of tumors, as it has been shown to promote several hallmarks of cancer *via* biochemical and biomechanical signaling. In this regard, melanoma cells are highly sensitive to ECM composition, stiffness and fiber alignment because they interact directly with the ECM in the tumor microenvironment *via* cell surface receptors, secreted factors or enzymes. Importantly, seeing as the ECM is predominantly deposited and remodeled by myofibroblastic stromal fibroblasts, it is a key avenue facilitating their paracrine interactions with melanoma cells. This review gives an overview of melanoma and further describes the critical roles that ECM properties such as ECM remodeling, ECM-related proteins and stiffness play in cutaneous melanoma progression, tumor cell plasticity and therapeutic resistance. Finally, given the emerging importance of ECM dynamics in melanoma, future perspectives on therapeutic strategies to normalize the ECM in tumors are discussed.

## Introduction

Early-stage cutaneous melanoma (clinical stage I – II) that has not presented with nodal or distant metastasis is effectively treated with surgery (1). However, late-stage cutaneous melanoma (clinical stages III and IV) can present with metastases in the lymph nodes, microsatellite and in-transit metastatic lesions (stage III) or it can disseminate to distant organs (stage IV) (1). It is therefore concerning that approximately 60% of melanoma patients present with metastatic lesions at the lymph nodes and 15% of patients experience metastases at distant organs including the lungs, liver, brain or bone (2, 3). Advanced melanoma is currently treated with chemo- and more recently, with immuno- and targeted therapies, however, it is often recalcitrant to these treatment regimens (2). Prognosis is poor for patients with advanced melanoma, as approximately 50-60% of these patients acquire drug resistance and go into therapeutic failure (3, 4). A possible reason for this, is that melanomas are highly heterogeneous within single tumors but also amongst different tumors at metastatic sites (5). This heterogeneity is in part caused by non-genetic mechanisms, which can drive melanoma cells to readily and reversibly switch between different phenotypic states (6, 7). The tumor microenvironment (TME) critically affects melanoma progression and tumor cell states at both the primary and secondary sites and has been shown to contribute to therapeutic resistance (8, 9).

The TME, which has been recognized as a hallmark of cancer, is a complex and evolving milieu that consists not only of tumor cells but also consists of non-malignant cell types, including fibroblasts, immune cells, nerve cells and endothelial cells, as well as the extracellular matrix (ECM) (10). Thus, the crosstalk between melanoma cells and their cellular and non-cellular surroundings plays a key role in disease progression and impacts the efficacy of anti-cancer therapies (6). The ECM is an important feature of the melanoma TME because ECM-derived biochemical and biomechanical cues have been shown to influence melanoma progression, tumor cell plasticity and therapeutic resistance (11, 12). The ECM is rich in proteins that form a 3D-meshwork of varying alignment and stiffness. Importantly, in cancers including melanoma, the ECM is drastically remodeled resembling fibrosis, especially during invasion and in response to therapies (12–15). This review begins with an overview of melanoma, including disease progression, current therapies and phenotypic plasticity, followed by a description of how ECM architecture is modified in the melanoma TME. Finally, we discuss the role of ECM signaling in melanoma progression, phenotypic plasticity and therapeutic resistance and give future perspectives on potential therapeutic strategies to normalize the melanoma TME.

## Overview of melanoma

### Melanoma progression

Cutaneous melanoma arises from neural-crest derived pigment producing melanocytes in the epidermis that have undergone genetic alterations, leading to aberrant growth and clonal expansion (16). While some melanomas arise from pre-existing naevi, the majority of melanomas arise *de novo* (17). The Clark model of cutaneous melanoma progression is a classical and well accepted model based on histopathological analyses of melanoma tissue samples at various stages of progression (18). This model begins with aggregated melanocytes forming a benign nevus in the epidermis that experience abnormal growth to form a dysplastic nevus, which can progress, upon clonal expansion, to the radial growth phase (RGP), where the melanoma cells are confined to the epidermis (18). Should the melanoma cells proliferate further and breach the basement membrane, they progress to the vertical growth phase (VGP), as the melanoma cells are able to invade the dermis (18). At the molecular level, melanocytic cells in the benign nevus, frequently have oncogenic mutations in the *BRAF* or *NRAS* genes. Furthermore, these lesions undergo dysplasia due to the loss of tumor suppressive signaling and the loss of expression of key cell cycle regulators. For example, the progression from a dysplastic nevus to the RGP occurs as a result of unchecked mutations and epigenetic changes in cell cycle and cell survival regulators including *PTEN*, *NF1*, *CDKN2A*, and *CCND1* that allow the melanoma cells to bypass senescence (18–20). Additionally, approximately 15% of melanomas occur due to a familial genetic predisposition, resulting in germline mutations in *CDKN2A* (most common) *CDK4*, *TERT*, *ACD*, *TERF2IP*, *POT1*, *MITF*, *MC1R*, and *BAP1* (21). The transition from the RGP to the VGP is characterized by an upregulation of mesenchymal markers such as N-cadherin, secreted protein acidic and cysteine rich (SPARC) and β-catenin, as well as the downregulation of the cell-cell adhesion molecule E-cadherin (18, 22, 23). Once the melanoma cells have invaded the dermis they can disseminate *via* lymphatic or blood vessels and metastasize to distant organs, which is considered the metastatic phase (18).

Although the Clark model has often been used as a reference for melanoma progression in studies investigating the molecular mechanisms that are driving melanoma progression, it is not routinely used for clinical melanoma diagnosis and classification. The first melanoma classification used by clinicians is the Breslow index, which measures the thickness of the tumor because a direct correlation exists between the depth of the melanoma in the skin and patient survival (24). Furthermore, the American Joint Committee on Cancer (AJCC) has recommended the use of the TNM (Tumor, Node, Metastasis) classification (stages 0 – IV), which grades the melanoma according to the thickness, the presence of ulceration in biopsies and any evidence of metastatic dissemination or lesions (25). More recently following the analysis of RNA, DNA and protein in 333 primary or metastatic samples from 331 melanoma patients, The Cancer Genome Atlas Network has proposed the classification of melanoma according to genetic mutations into 4 categories: BRAF, NRAS, NF-1 and triple negative (wild type) (20). The data indicated that mutations in *BRAF* are prevalent in 50% of melanoma patient samples, followed by *NRAS* (30%) and *NF1* (14%) and 15% of melanomas did not harbor somatic mutations in *BRAF, NRAS* or *NF1* (20).

### Current therapies for melanoma treatment

Current FDA approved therapies for advanced melanoma include chemo-, immuno- and targeted therapies (2). The most commonly used chemotherapeutic drug used for melanoma treatment is Dacarbazine, which was initially approved for melanoma in the 1970s (26). However, as a monotherapy it was shown to have minimal efficacy in treating advanced melanoma, with the average response rate in patients being less than 5% and the 5 year survival of patients was suggested to be less than 6% (26–28). Furthermore, the combination of Dacarbazine treatment with earlier immunotherapies such as Interferon -2β and Interleukin-2 showed very little improvement in patient survival (2, 28). Notably, the landscape of advanced melanoma treatment has changed drastically in the last decade, following the FDA-approval of immune checkpoint/blockade inhibitors and targeted therapies (4, 29). It is important to note that the genetic classification of melanomas has become particularly important in determining whether a patient is eligible for immuno- or targeted therapy, as the administration of either immuno- or targeted therapy depends on the *BRAF* status (2). Should the patient have wild type *BRAF*, they are treated with immune check-point inhibitors and if a V600E/K mutation is identified in *BRAF*, they can be treated with BRAF and MEK inhibitors or with immunotherapy (2, 26).

Immunotherapy involves the administration of immune checkpoint inhibitors, which are antibodies against cytotoxic T-lymphocyte-associated antigen 4 (CTLA-4) and programmed cell death protein 1 (PD-1) receptor/PD-1 ligand (PD-L1) (2, 30). Mechanistically, the anti-CTLA-4 antibody binds to the inhibitory checkpoint CTLA-4 receptor that is present on the surface of regulatory T-cells, which prohibits the interaction of T-cells with antigen presenting cells that express the CTLA-4 ligand, B7 co-stimulatory molecule (31). The binding of anti-CTLA-4 antibodies to CTLA-4 promotes the activation of T-cells and the inhibition of the immune checkpoint blockade (30, 31). Activated T-cells are then able to illicit an immune response *via* cytokine secretion, clonal expansion and infiltration into the tumor leading to tumor regression (30, 31). To date, Ipilimumab is the only FDA approved anti- CTLA-4 antibody for the treatment of malignant melanoma and is used in clinics as a monotherapy or in combination with the anti-PD-1 antibody Nivolumab (2, 30). T-cell activation can also be suppressed *via* another receptor-ligand interaction, whereby ligands PD-L1 and PD-L2 can bind the PD-1 receptor, resulting in a co-inhibitory effect (2, 30). The anti-PD-L1 antibody prevents the binding of PD-L1 or PD-L2 to the PD-1 receptor, thereby promoting T-cell activation (32). The PD-1 receptor is expressed on the surface of immune cells including T-cells. Melanoma cells express the ligands PD-L1 and PD-L2 and fibroblasts present in the TME were shown to express the ligand PD-L2 (33, 34). In 2014, Nivolumab became the first FDA-approved anti-PD-1 antibody for the treatment of unresectable or metastatic melanoma and subsequently, Pembrolizumab was also approved by the FDA for malignant melanoma treatment in 2015 (2, 28, 30). Currently, Nivolumab and Pembrolizumab are administered as monotherapies or Ipilimumab can be administered in combination Nivolumab with as a combination therapy (2, 28, 35). Although immunotherapies have improved overall and relapse free survival in patients with late stage (stage III and IV) melanoma, immune-related adverse effects are particularly common in these patients including diarrhea, fatigue, skin rash, nausea, headaches and joint pain (2).

BRAF and MEK inhibitors, target the mitogen-activated protein kinase (MAPK)/ERK signaling pathway which is constitutively active in *BRAF* mutant melanoma (2, 30). *BRAF* encodes the rapidly accelerated fibrosarcoma (RAF) serine/threonine kinases (isoforms: ARAF, BRAF and CRAF) that constitutively dimerize due to the V600E mutation (36). Seeing as BRAF and its isoforms are upstream of the MAPK signaling cascade, they amplify its constitutive activation (37, 38). The MAPK signalling cascade has been shown to play a role in key processes of melanomagenesis such as differentiation, survival, proliferation, metastasis, invasion and angiogenesis (36). Briefly, this signaling pathway is activated by extracellular signals binding to G-coupled receptors or receptor tyrosine kinase (RTK) at the cell membrane, which then activates intracellular GTPase NRAS (also KRAS and HRAS) to NRAS-GTP that is able to further activate all 3 RAF protein isoforms, including BRAF (37, 39). This results in the phosphorylation of downstream MAP/ERK kinases (MEK) and the initiation of the MAPK signaling cascade by further phosphorylation of ERK1/2, which can induce transcriptional and translational signaling (37, 39). Vemurafenib was the first selective oral BRAF small molecule inhibitor, approved in 2011 by the FDA, for advanced melanoma treatment and in 2013 a second selective BRAF inhibitor, Dabrafenib, was approved by the FDA (2, 39). Furthermore, the MEK inhibitor Trametinib became FDA-approved in 2013. In clinical trials Trametinib was also partially successful in treating melanomas harbouring NRAS mutations (2, 40). Vemurafenib, Dabrafenib and Trametinib can all be administered as monotherapies and Dabrafenib and Trametinib can also be administered as a part of BRAF and MEK inhibitor combination therapy (2, 4). Furthermore, in 2015, the FDA approved the oral administration of the MEK inhibitor Cobimetinib in combination with Vemurafenib, as a combination therapy (2, 38).

Seeing as these immuno- and targeted therapies significantly improved overall response in approximately 40% of melanoma patients, they are fast becoming the standard of care for unresectable or metastatic melanoma in many countries including the United Kingdom, the United States, Australia and in Europe (2, 30). Approximately 50-60% of melanoma patients treated with the current FDA-approved immuno- and targeted therapies respond transiently to treatment but unfortunately often progress to develop therapeutic resistance (4, 29). It is therefore not surprising that the resistant mechanisms employed by melanoma cells to circumvent these treatments constitute a major research area in the field of melanoma (3, 41).

### Melanoma plasticity

It is fast becoming accepted that non-genetic heterogeneity is a key feature of melanomas that contributes to tumor progression and therapeutic resistance (3, 6, 41). Non-genetic heterogeneity is characterized by melanoma cell plasticity, as it has been shown that within a tumor. In order to adapt to changing pressure and various conditions in the TME, melanoma cells exist in different phenotypic states and can readily switch between distinct transcriptional programs, which are suggested to be distinct for different phenotypic states (3, 6, 41). For example, some melanoma cells might be highly differentiated, more proliferative and melanocytic, whereas some cells might be in a slow-cycling dedifferentiated and more invasive mesenchymal-like state (41). Phenotypic switching of melanoma cells has been shown to occur as a result of reversible epigenetic changes caused by but not limited to chromatin remodeling, microRNAs, long non-coding RNAs and histone modifications (3, 41). These transcriptional and epigenetic changes are thought to occur in melanoma cells as an adaptive response to cues in the TME such as hypoxia, glucose or amino acid deprivation, secreted and inflammatory factors, ECM mechano-signaling and in response to immuno- and targeted therapies (3, 7, 41, 42). In response to these cues, melanoma cells are able to temporarily reprogram their phenotype with regards to their proliferation, cell cycling, metabolic status and motility (7, 41).

To date, melanoma cells have been described to exist in several phenotypic states including the differentiated proliferative and melanocytic, the dedifferentiated invasive and mesenchymal-like, the dedifferentiated neural crest stem cell-like, starved, as well as transitory states (43–47). The melanocytic and invasive mesenchymal-like states were identified in melanoma patient tumors using RNA sequencing (44). Single-cell RNA sequencing further identified the differentiated, starved, neural crest-like states, as well as the melanocytic (more proliferative) and mesenchymal-like (more invasive) states in primary melanoma cells that were isolated from biopsies of melanoma patients, who were treated with BRAF or MEK inhibitors (45, 48). These phenotypic states have been linked to distinct transcriptomic profiles and gene signatures in melanoma cells (43–45, 47, 49, 50). More specifically, changes in the expression of the master regulator microphthalmia-associated transcription factor (MITF) has been associated with melanoma phenotypic switching (41, 49, 51).

### Molecular signatures associated with drug resistant melanoma phenotypes

MITF is a key transcription factor that regulates melanocyte development, pigmentation and melanoma progression and has been implicated in cellular processes such as differentiation, survival, cell cycle regulation, senescence bypass, autophagy and lysosomal production and regulation (52). Furthermore, MITF plays a role in genetic processes such as DNA damage repair and chromosome stability (52). Interestingly, MITF was recently shown to directly repress genes associated with the expression of ECM and focal adhesion pathways, demonstrating its involvement in regulating cell-ECM interactions (53). With regards to melanoma phenotypic states, MITF^high^ expressing melanoma cells have been associated with a more differentiated, melanocytic and proliferative state, whereas MITF^low^ expressing melanoma cells have been characterized as more aggressive, dedifferentiated and mesenchymal-like and were found to be associated with invasion and therapeutic resistance (44, 49, 51).

A second transcriptomic signature that has been associated with the melanoma cell phenotypic switch that is inversely correlated to MITF expression, is the expression of the receptor tyrosine kinase AXL (50, 54). This receptor is expressed by cancer cells including melanoma cells and immune cells (55). The binding of AXL and its ligand growth arrest-specific protein 6 (GAS6) induces intracellular signaling that can affect cancer progression, metastasis and drug resistance (55). In melanoma, compared to the MITF^high^AXL^low^ signature, which promotes a more proliferative and drug sensitive phenotype, the MITF^low^AXL^high^ signature is specifically associated with an invasive, mesenchymal-like phenotype (50, 54). Importantly, MITF^low^AXL^high^ expression was shown to be common in *BRAF* mutated melanoma and has been identified in patients who relapsed following BRAF and MEK inhibitors treatment, thereby indicating that this signature results in a drug resistant phenotype (50).

Melanoma cells that have the MITF^low^AXL^high^ signature, which are associated with a more dedifferentiated phenotype can express epithelial to mesenchymal transition (EMT) molecular markers (3, 41, 56). It is important to note that melanoma cells cannot undergo EMT as melanocytes arise from the neuroectoderm but melanoma cells can be reprogrammed to mesenchymal-like cells (56, 57). In this regard, MITF/AXL expression was shown to correlate with SOX10 and SOX9 transcriptional signatures, which are molecular markers of epithelial-like and mesenchymal-like phenotypes, respectively (41). Therefore, MITF^low^AXL^high^ expressing melanoma cells have been associated with SOX10^low^SOX9^high^ expression and the opposite was shown for MITF^high^AXL^low^ expressing cells. Furthermore, lower levels of SOX10 have been shown to contribute to therapeutic resistance, in part, *via* the transcription factors JUN and/or AP-1 and TEAD (47, 58). The SOX10^low^SOX9^high^ signature has also been shown to induce a mesenchymal-like phenotype in melanoma cells with the increased expression of mesenchymal markers such as vimentin, platelet derived growth factor receptor-β (PDGFRβ) and α-smooth muscle actin (αSMA) (46, 59–61). Importantly, this signature has also been associated with drug resistance (12, 14).

Additionally, the HIPPO pathway, in particular the transcriptional co-activator yes-activated protein (YAP)-mediated signaling, has been implicated in the dedifferentiated, invasive mesenchymal-like phenotype (14, 41, 56). Accordingly, YAP and its downstream TEAD signature have been shown to be associated with melanoma metastasis and invasion (62–65). Importantly, there is also some evidence that in melanoma cells, the nuclear accumulation of the two transcriptional co-activator paralogs and mechanotransducers YAP and TAZ promotes resistance to BRAF inhibitors (65). More specifically, in MITF^low^AXL^high^ melanoma cells that were resistant to the BRAF inhibitor Vemurafenib, increased actin stress-fiber formation and remodeling were observed, which were shown to be dependent on increased levels of nuclear YAP/TAZ (65). Furthermore, the pharmacological inhibition or knockdown of YAP in BRAF inhibitor resistant melanoma cells decreased ERK1/2 signaling, remodeling of the actin cytoskeleton and tumor growth and therefore enhanced BRAF inhibitor efficacy (14, 66). Importantly, this adaptive phenotypic transition of melanoma cells towards a dedifferentiated state, associated with upregulated expression of genes involved in EMT, ECM reprogramming, wound healing, cytoskeletal remodeling and YAP/TAZ/TEAD signatures has also been linked to immune evasion and resistance to PD-1 blockade immunotherapy (67, 68).

Interestingly, melanoma cells in a highly differentiated and melanocytic state, characterized by MITF^high^, expression have also been shown to play a role in resistance to BRAF and MEK targeted therapies, *via* the rheostatic (on/off) regulation of the transcription factors, PAX3 and BRN2 (51, 69). Furthermore, it has been observed that BRAF inhibitor treatment led to the enrichment of a small population of melanoma cells in a dedifferentiated state, termed BRAF inhibitor persister cells. These persister cells constitutively expressed the Aryl hydrocarbon Receptor (AhR) transcription factor and were shown to promote relapse (21, 70). In addition, another population of persister melanoma cells was recently described to account for minimal residual disease (MRD), as this cell population had acquired tolerance to MAPK inhibitors *via* non-genetic mechanisms. This distinct phenotype was shown to be a transient neural crest stem cell (NCSC) population, which was shown to be dependent on the glial cell line-derived neurotrophic factor cascade that consequently resulted in AKT driven survival *via* focal adhesion kinase (FAK) signaling (71). To this end, the evidence for the role of phenotypic switching by melanoma cells, as an adaptive process involved in non-mutational resistance, is convincing but the investigation into other transitory phenotypic states and the factors that influence and regulate this phenotypic switching is ongoing (70). Given that melanoma cells can readily switch between these different phenotypic states in response to external cues from the TME or therapeutic insults, it is critical to improve our understanding of the biochemical and biomechanical signaling between melanoma cells and the tumorigenic ECM.

## ECM architecture modification and therapeutic resistance

### Key players in ECM modification

A major cause of altered ECM organization and dynamics during cancer is dysregulated ECM synthesis and remodeling by fibroblasts that have differentiated into cancer-associated fibroblasts (CAFs). ECM remodeling in the TME and organ fibrosis display striking similarities and are both considered to be dysregulated wound healing processes (12). CAFs are the predominant cells in the stroma that modify ECM architecture using several ECM remodeling mechanisms (72). Importantly, CAFs organize and rigidify the ECM by exuding mechanical and tractional forces, resulting in the deposition of ECM fibers that are radially aligned in a parallel fashion (73, 74). CAFs are activated fibroblasts that can originate from resident fibroblasts but may also arise from mesenchymal stem cells, endothelial cells and epithelial cancer cells that have undergone EMT (72, 75, 76). To date, there is no unique marker by which CAFs can be identified, as CAFs express a combination αSMA, fibroblast activation protein-α (FAPα), β_1_integrin (CD29), fibroblast specific protein 1 (S100A4), caveolin 1, podoplanin and PDGFRβ (72, 76, 77). Furthermore, CAFs can express various combinations of these markers and at different levels (78). Given the numerous cells of origin of CAFs and their expression of various markers, it is not surprising that the CAF population in the TME is heterogeneous (72). Several studies have identified two key CAF phenotypically distinct clusters, inflammatory CAFs (iCAFs) and myofibroblastic CAFs (myCAFs), in the TME of breast cancer, pancreatic adenocarcinoma and melanoma (78–82). myCAFs exhibit myofibroblastic properties that are associated with increased levels of αSMA expression, ECM remodeling, actin-myosin adhesion, wound healing and transforming growth factor β (TGFβ) and interferon αβ (IFNαβ) signaling pathways (79, 82–84). Furthermore, myCAFs were found to be located in the vicinity of cancer cells suggesting that paracrine signaling between myCAFs and cancer cells is critical for ECM remodeling in the TME (78, 82).

In cutaneous melanoma, melanoma-associated fibroblasts (MAFs) are primarily responsible for depositing and remodeling ECM in the TME (85). Interestingly, in aged skin, ECM architecture was shown to be modified to a more aligned organization by aged fibroblasts that produced less of the hyaluronic and proteoglycan link protein, HAPLN1. This aligned ECM in aged skin was shown to promote metastasis and influence therapeutic response and immune cell motility in the melanoma TME (86). ECM remodeling and fiber alignment by MAFs therefore play an important role in melanoma cell invasion and migration, immune escape, metastasis, intra-tumoral heterogeneity and therapeutic resistance (14, 56, 87–90).

ECM stiffness and fiber alignment in solid tumors, including breast carcinoma, sarcomas and melanoma, contribute to increased interstitial pressure, which results in blood vessel collapse and impaired blood supply, leading to hypoxic conditions (91). Importantly, variations in ECM biophysical properties and mechanical forces have been described to alter cellular metabolism in cancer cells (92, 93). Importantly, immunohistochemical analyses of primary and metastatic melanoma patient samples in a tissue microarray, showed that molecular markers of collagen levels were associated with the transcriptomic signature for melanoma cell phenotypic dedifferentiation *via* the YAP/PAX3/MITF axis (90). Furthermore, this dedifferentiated phenotype was found to be associated with increased collagen fiber abundance and correlated with poorer survival of melanoma patients (90).

Interestingly, we recently showed that melanoma cells that have the specific MITF^low^AXL^high^ transcriptomic signature and a dedifferentiated, invasive and mesenchymal-like phenotype, display the ability to autonomously deposit and remodel the ECM, resulting in a dense collagen rich and highly organized (aligned fibers) stiff ECM (14). Furthermore, mass spectrometry was used to compare the biochemical composition of 3D-ECM deposited by dedifferentiated, invasive and mesenchymal-like (MITF^low^AXL^high^) or differentiated and melanocytic (MITF^high^AXL^low^) melanoma cells. Matrices derived from dedifferentiated, melanoma cells were found to be enriched for key ECM structural proteins (e.g., fibronectin, laminin, collagen I, collagen IV, collagen VIII, tenascin) and proteins involved in ECM remodeling processes (e.g., Thrombospondin-1, Lysyl oxidase homolog 2 (LOXL2), ADAMTS-like protein 1 and Plasminogen activator inhibitor 1) (14).

MAFs and dedifferentiated mesenchymal-like melanoma cells can produce and remodel the ECM in response to cues from the TME such as secreted factors (e.g., TGFβ, PDGF, fibroblast growth factor 2), hypoxia, ECM mechanical signals and in response to BRAF inhibitor treatment (85). On the other hand, melanoma cells adhere to the ECM and interact directly with the ECM *via* cell surface receptors. Melanoma cells are therefore highly sensitive to the presence of specific ECM proteins, the alignment of ECM fibers and the mechanical stiffness of the ECM, which can all induce intracellular signaling and metabolic reprogramming, thereby altering melanoma cell behavior (3, 13, 14, 62). Furthermore, melanoma cells have been shown to reprogram fibroblasts in the lymph node to modify the biochemical properties and architecture of the ECM in the lymph node. This reprogramming of reticular fibroblasts is important for melanoma cell invasion at the metastatic site which is often the lymph node (94, 95). Thus, the role of the ECM in melanoma progression and therapeutic resistance resembles a feedback loop, which involves the interplay between fibroblasts, melanoma cells and ECM biochemical and biomechanical properties.

### ECM remodeling mechanisms

ECM remodeling leads to alterations in ECM composition, fiber organization and stiffness that can influence melanoma progression, phenotypic switching and therapeutic response (96). The ECM is remodeled *via* three key mechanisms: i) ECM deposition and post translational modification, ii) degradation and iii) force-mediated ECM modification (97). The protein-rich ECM consists of approximately 300 macromolecules that are termed the core matrisome (98). Key matrisome components include collagens (e.g., collagen I, collagen IV), glycoproteins (e.g., fibronectin, laminin, elastin, tenascin), proteoglycans which are glycoproteins that have attached glycosaminoglycans (e.g., hyaluronan, perlecan). Furthermore, matrisome associated proteins include ECM-bound growth and secreted factors (e.g., vascular endothelial growth factor, hepatocyte growth factor, PDGF) and matricellular proteins (e.g., SPARC) (98, 99). ECM composition is dependent on the deposition and post-translational modification of ECM proteins (97).

During ECM deposition and post translational modification, ECM proteins are synthesized and translocated to the Golgi in the cytoplasm, where further post translational modifications can occur (97). For example, collagen is first synthesized as pre-collagen and is post translationally modified in the Golgi to a procollagen α-chain by glycosylation, pro-peptide alignment, disulphide bond formation and hydroxylation (97). Hydroxylation of the procollagen α-chain by lysyl hydroxylases results in triple helix formation of procollagen α-chains, which is followed by the secretion of procollagen α-helices into the extracellular space (97). In the extracellular space, collagen fibrils are formed by proteolytic cleavage of pro-peptides at the C and N terminals (97). Thereafter, the collagen fibrils are assembled *via* covalent cross-linking by lysyl oxidase (LOX) family members (97). For example, LOXL2 secreted by CAFs, crosslinks collagen and elastin fibers (100). The cross linking of ECM fibrils, in particular collagen fibers, is important for fiber assembly and also increases the tensile strength and stiffness of the ECM (97). Furthermore, collagen fibers are also cross linked to other ECM fibers such as fibronectin fibers by tissue transglutaminase 2, as the deposition of collagen I and III is dependent on the presence and stability of deposited fibronectin (97).

The second mechanism by which the ECM is modified is degradation, which involves the proteolytic cleavage of ECM components by proteases such as matrix metalloproteases (MMPs) (101). Proteolytic cleavage of ECM components promotes the release of ECM fragments, growth factors and cytokines in the ECM milleu, which promotes migration and infiltration of cells in the TME (102). ECM degradation therefore plays an important role in the crosstalk between the ECM, melanoma cells, MAFs and other cells present in the TME such as immune and endothelial cells (96, 97).

The third mechanism by which the ECM is remodeled, is force-mediated modification (96, 97). This involves the binding of cellular receptors such as integrins to ECM molecules including collagens and fibronectin. This leads to traction and mechanical forces, which are transduced to ECM molecules *via* integrins (96, 97). This results in conformational changes that expose binding sites on ECM molecules, which can then self-assemble into fibrils and create an ECM with aligned and parallel fibers (97). Seeing as melanoma cells interact directly with the ECM *via* cell membrane receptors at each stage of melanoma progression, ECM remodeling is tightly regulated during melanomagenesis (13). However, the role of ECM-derived mechanical signaling, which is called mechanotransduction, in melanoma biology is not entirely understood and is currently a dynamic and growing topic in the field.

### ECM-mediated signaling in melanoma progression

ECM architecture has been shown to play important roles in melanoma phenotypic switching, metastasis and therapeutic resistance because ECM composition, alignment and stiffness directly affect intracellular signaling (96, 97, 103). These intracellular signaling cascades alter melanoma cell behavior and occur as a result of direct binding with the ECM *via* transmembrane receptors, including integrins and discoidin domain receptors (DDRs) (102). In this regard, fibrillar collagen I and non-fibrillar collagen IV, as well as fibronectin, play important roles in melanoma cell adhesion and migration, thereby promoting metastasis and invasion (13, 102). Melanoma cells were shown to directly interact with collagen IV in the TME *via* integrins (α_2_β_1_ and α_3_β_1_), which promoted melanoma cell spreading and motility, through the activation of MMP2 and MMP9 (104–107). Seeing as collagen IV is a found in basement membranes, integrin-mediated interaction of melanoma cells with collagen IV is necessary for melanoma cells to breach the basement membrane when progressing from the RGP to the VGP, where they invade into the dermis prior to disseminating to other organs *via* lymphatics and blood vessels (108, 109). Integrins have also been described to play an important role in melanoma cell adhesion to other ECM components including laminin, which is also found in basement membranes and fibronectin (108, 110). For example, integrins (α_4_β_1_ and α_5_β_1_) were found to be involved in promoting melanoma cell attachment to fibronectin fibers which promoted migration of melanoma cells (108).

The direct interaction of melanoma cells with fibrillar collagen I *via* DRR1 and DDR2 has also been shown to contribute to melanoma cell migration and invasion (111–113). For example, one study showed that in murine melanoma cells, the interaction of DDR2 with collagen I resulted in increased expression of MMP2 and MMP9 *via* the intracellular ERK/NF-κB signaling pathway and promoted melanoma cell invasion (112). Furthermore, the interaction of cancer cells with aligned ECM fibers promoted directional migration of cancer cells (102, 114). In this regard, melanoma cells were found to mimic the directional migration of fibroblasts along collagen fibrils in a 3D collagen ECM model (114, 115). Melanoma cells were described to elongate and migrate in parallel to collagen fibrils by extending actin-rich protrusions, called filapodia, which adhere to and detach from collagen fibrils in cycles, as the melanoma cells are migrating (115). Furthermore, fibronectin was found to be an essential ECM adhesive component required for the directional migration of pancreatic, prostate and head and neck squamous carcinoma cells (116). The fibronectin monomer consists of 15 domains and two extra domains and the extra domain A (EDA) has also been implicated in the activation of TGFβ signaling and its presence in tumors has been associated with increased ECM remodeling, MMP expression and actin cytoskeleton re-organization (117).

Interestingly, melanoma invasion is affected by both ECM fiber alignment and ECM stiffness, as the shape of melanoma cells was shown to change in response to stiffer and more fibrous substrates (118). For example, the elongation of melanoma cells along aligned collagenous fibers was observed and melanoma cell polarization was found to be directly proportional to collagen matrix fiber alignment (118). Several other studies support these findings, as aligned matrices were shown to promote carcinoma, pancreatic cancer and ovarian cancer cell elongation and invasion (87, 119, 120). Furthermore, ECM fiber alignment and stiffness have been reported to be critical for breast cancer invasion, which is reviewed in detail by Kai et al, 2019 (102, 121–123). Surprisingly in contrast to these studies, experiments using varying ECM stiffness models, suggested that the correlation between melanoma cell invasion and substrate stiffness might differ to what was observed for other cancer models. Melanoma cell invasion was shown to be restricted by both soft and highly stiff collagen substrates, suggesting a non-linear relationship between substrate stiffness and optimum invasion (14, 124). These findings are further supported by another group which showed that collagen matrices of an intermediate stiffness provided the optimal conditions for facilitating melanoma cell invasion (118). These differences might be related to the tissue origin of the breast cancer and melanoma cells. Breast tissue is softer than the skin because breast tissue differs to the skin in term of its rigidity and elasticity. Thus, during malignancy, breast cancer cells initially progress in a softer matrix associated with the physiological breast tissue stiffness but require a stiffer matrix for invasion, whereas melanoma cells initially progress in a stiffer environment in the skin and require a softer and less dense matrix for invasion (125, 126).

It is important to note that the studies discussed in this section employed *in vitro* (e.g., hydrogels or decellularized matrices) or animal models to investigate the role of ECM alignment and stiffness in melanoma progression, in particular during melanoma cell migration and invasion. These findings have yet to be confirmed in clinical melanoma samples.

### ECM architecture influences responses to targeted- and immuno-therapies

ECM stiffness and alignment, caused by collagen abundance and crosslinking in the TME, have been shown to contribute to melanoma cell phenotypic switching and therapeutic resistance by inducing mechanosignaling, which can alter cellular behavior to promote a mechanosensitive and resistant mesenchymal-like phenotype (14, 90). Collagen abundance and stiffness were shown to promote the nuclear localization of YAP, which regulated melanoma cell pigmentation and differentiation through the expression of MITF (90). Moreover, one study suggested that higher MITF levels in melanoma cells might be associated with the repression of ECM and focal adhesion genes (53). The effect of MITF repression of these genes was found to be reversible when MITF was knocked down in melanoma cells, resulting in increased focal adhesion complexes and resistance to BRAF inhibitor treatment (53). Interestingly, our group demonstrated that stiffer collagen substrates resulted in increased in mechanosensing *via* YAP and myocardin-related transcription factor (MRTF) in MITF^low^, dedifferentiated, invasive mesenchymal-like melanoma cells, in response to BRAF inhibitor treatment (14). Furthermore, this mechanosensitivity promoted melanoma cells to deposit a stiff, organized and drug-protective ECM and these observations were further confirmed *in vivo* (14).

We also recently demonstrated that stiff and aligned collagen and fibronectin rich matrices deposited by MAFs protected melanoma cells from BRAF/MEK inhibitor treatment, when melanoma cells were cultured on top of these matrices (127). This matrix-mediated drug resistance was demonstrated to be DDR1 and DDR2 dependent and promoted melanoma cell survival *via* the NIK/IKKα/NF-κB2 signaling pathway (127). Therefore, the alignment of fibrils and ECM composition plays an important role in melanoma cell migration and contributes to melanoma cell escape mechanisms to targeted therapies. These studies therefore suggest that in melanoma cells, ECM topography plays important roles in MITF levels and therefore contribute to phenotypic switching and drug adaptive responses.

The induction of ECM-related signaling pathways is a mechanism that is implored by melanoma cells to evade BRAF inhibition and confers tolerance and resistance to BRAF inhibitor treatment (53, 71, 88, 127, 128). For example, one study described upregulated expression of the collagenase and metalloproteinase MT1-MMP in melanoma cells, which acquired resistance to the BRAF inhibitor Vemurafenib (128). Furthermore, this resistance mechanism involved β_1_integrin/FAK signaling and was also dependent on the TGFβ cascade (128). Importantly, the inhibition of this ECM signaling cascade, using the MT1-MMP selective inhibitor ND322 restored melanoma cell sensitivity to BRAF inhibitor treatment (128). Interestingly, in PDX models treated with MAPK inhibitors, a subpopulation of drug-tolerant melanoma cells was described to be associated with the emergence of a transient neural crest stem cell (NCSC) state. The NCSC state was a persister state that most closely corresponds to the minimal residual disease (MRD) observed in clinics and displayed increased activation of FAK-dependent AKT survival signaling (71). Delaying the onset of this non-genetic resistance was achieved by FAK inhibition and the consequential targeting of NCSC-like melanoma cells (71).

Interestingly, BRAF inhibitor treatment has also been implicated in activating MAFs to remodel the ECM making it stiffer, which induced drug tolerance in melanoma cells (88). Furthermore, the direct interaction of melanoma cells *via* β_1_integrin with the ECM deposited by MAFs, induced intracellular FAK signaling that consequently re-activated the ERK signaling pathway in melanoma cells, following BRAF inhibitor treatment (88). Importantly, a fibroblastic and remodeled ECM was observed in the tumoral stroma of melanoma tissue from Vemurafenib (BRAF inhibitor) treated patients (88). Additionally, myCAF (myofibroblastic MAF) clusters with distinguished ECM and TGFβ signaling signatures have been associated with primary resistance to immunotherapies in samples from non-responder melanoma patients (81).

These studies therefore provide some insight into the contribution of ECM signaling and remodeling in response to targeted- and immuno-therapies, in melanoma. However, these ECM-related resistance mechanisms employed by melanoma cells appear to be multifaceted and warrant further investigation.

## Strategies to normalize the TME

Given that MAFs are the primary cells that remodel the ECM in the TME and that ECM composition, alignment and stiffness have been shown to promote metastasis, immunosuppression and therapeutic resistance, an obvious strategy to counteract this effect is to target MAF functions in the melanoma TME. However, it was reported that ablating CAFs in pancreatic ductal adenocarcinoma (PDAC) mouse models resulted in invasive, dedifferentiated and highly hypoxic tumors (129). Furthermore, evidence of EMT and cancer stem cells were observed in tumors, which resulted in poor patient survival and immune suppression. Additionally, the authors also reported that lower myofibroblast number in PDAC patient tumors correlated with reduced patient survival (129). Interestingly, another study showed that targeting iCAFs by JAK inhibition shifted them to a myCAF phenotype *in vivo* (130). In contrast, another group reported that the blockade of CAF contractility by the JAK1/2 inhibitor, Ruxolitinib, counteracted CAF-dependent carcinoma cell invasion and ECM remodeling (131). Thus, the issue of functional complexity and phenotypic heterogeneity of CAFs must be taken into consideration for the development of effective anti-CAF therapies (78). Therefore, the current opinion in the field is to rather achieve normalization in the TME than completely ablate CAFs (132, 133). This approach focuses on restoring homeostasis in the TME, in particular with regards to the ECM, to resemble a non-tumorigenic normal tissue-like state (132, 133). One possible way to achieve this could be to reprogram MAFs to a normal fibroblast phenotype. However, this requires improved understanding of the mechanisms underpinning the activation and functional diversity of MAFs in the melanoma TME.

In light of the current opinion to rather normalize the fibrotic-like tumorigenic ECM than ablate CAFs in the TME, it might be worthwhile to investigate whether the repurposing of anti-fibrotic drugs that are used to treat idiopathic pulmonary fibrosis, such as the TGFβ regulator Pirfenidone or the triple tyrosine kinase inhibitor Nintedanib, in combination with current therapies, might disrupt MAF signaling and ECM-mediated resistance (62). Another strategy might be to identify novel druggable targets in melanoma cells that promote the ECM remodeling abilities associated with the dedifferentiated, mesenchymal-like and resistant phenotype in melanoma cells. We recently identified a fibrosis-associated miRNA cluster (miR-143/-145) that was shown to play an important role in driving the ECM program in dedifferentiated, invasive and mesenchymal-like melanoma cells (15). Furthermore, we also found that, in an allograft melanoma model, the anti-fibrotic drug Nintedanib was efficient in preventing miR-143/-145 cluster upregulation in melanoma cells and in combination with MAPK targeting therapy, normalized the tumoral ECM niche and delayed relapse (15).

Another approach might be to disrupt ECM fiber linearization in the melanoma TME by targeting the YAP mechanotransduction pathway and DDR1/2-dependent collagen signaling using Verteporfin and Imatinib, respectively (134, 135). This treatment strategy might prevent targeted therapy-induced collagen linearization and ECM remodeling, thereby potentially abrogating the permissive stroma and improve drug response. Interestingly, a recent study found that collagen linearization can be reversed and consequently metastasis can be hampered by genetically or pharmacologically restoring levels of the secreted factor WISP2. WISP2 and WISP1 are matricellular proteins secreted by cancer cells (136). WISP1 binds to linearized collagen type I, whereas WISP2 inhibits the binding of WISP1 to collagen type I and in turn inhibits collagen linearization. The authors found that in cancer patients, *WISP2* expression was lower in solid tumors. Therefore, the restoration of a higher WISP2:WISP1 ratio, in an *in vivo* cancer model, normalized collagen fibers in the TME and drastically inhibited metastasis (136). Therapeutically disrupting ECM fiber linearization might therefore prove to be a valuable strategy in achieving ECM normalization in the melanoma TME.

Approaches to normalize the fibrotic-like ECM in the TME have also been described to improve response to immunotherapy (137). One study showed that, in isogenic murine cancer models, including melanoma, targeting DDR2, using the tyrosine kinase inhibitor Dasatinib, in combination with anti-PD-1, improved therapeutic response to immunotherapy (137). Furthermore, another group showed that targeting collagen stabilization (crosslinking) using a LOX inhibitor in combination with anti-PD-1 *in vivo*, not only reduced overall tumor stiffness but also allowed for improved T-cell migration in the melanoma TME, thereby improving the overall efficacy of this immunotherapy (138). Furthermore, in a preclinical melanoma study, mouse survival was prolonged when CAFs were targeted with Nintedanib in combination with anti-PD-1 treatment. Tumor growth was described to be repressed due to the disruption the fibrotic-like ECM, which led to improved infiltration of CD8+ T cells and the production of granzyme B, therefore improving anti-tumor immunity and response to immunotherapy in tumor-bearing mice (139). Another potential therapeutic strategy to normalize the TME was suggested by a group that investigated the combination of shPD-L1 loaded nanoparticles and the ECM degradation enzyme hyaluronidase in an *in vivo* melanoma model (140). The authors found that the hyaluronidase degraded the hyaluronic acid in tumors, thereby increasing the infiltration of the shPD-L1 loaded nanoparticles in the TME and resulted in improved PD-L1 gene silencing (140). Additionally, another group carried out an epigenetic screen to identify novel druggable targets for melanoma treatment and identified TP-472 (targets bromodomain-7/9) as a potential drug, as it effectively inhibited melanoma cell proliferation (141). Interestingly, transcriptome-wide mRNA sequencing revealed that TP-472 treatment downregulated genes encoding various ECM proteins, such as integrins, collagens, and fibronectins in melanoma cells (141). Therefore, these studies suggest that normalizing the fibrotic and dense ECM in the melanoma TME might facilitate improved immune cell infiltration and overall immune anti-tumor response, which has the potential to significantly improve the efficacy of immunotherapies and patient prognosis.

Interestingly, there is some evidence that ECM molecules can serve as biomarkers and can predict the efficacy of immunotherapy treatment in melanoma patients (142, 143). For example, one study described that higher levels of ECM and tissue remodeling proteins such as MMP degraded type III and type IV collagens, quantified in patient serum from metastatic melanoma patients prior to immune checkpoint inhibitor treatment, were predictors of poorer overall survival (143). Furthermore, another group recently demonstrated that levels of the ECM protein elastin microfibril interface-located protein 2 (EMILIN-2) vary amongst melanoma patients and that the absence of EMILIN-2 expression was shown to be associated with higher PD-L1 expression levels, that in turn, improved the efficacy of immunotherapy. A possible reason for this observation was suggested using an *in vivo* Emilin2-/- mouse model that showed improved vessel normalization and a decrease in hypoxia within tumors (142). Further investigation of ECM markers in melanoma patient serum might therefore be an interesting avenue to further explore, in order to identify novel melanoma prognostic biomarkers.

The normalization of the melanoma TME therefore has the potential to limit immune suppression, increase therapeutic responses and improve advanced melanoma patient prognosis. However, novel therapeutic strategies to prevent or reverse the altered ECM within tumors require further investigation.

## Conclusion

The ECM is a dynamic feature of the TME that can dramatically influence cancer biology. Herein, we reviewed how melanoma ECM topology is remodeled by MAFs and dedifferentiated, invasive and mesenchymal-like melanoma cells. These remodeling changes result in ECM architectural modifications including changes in ECM deposition, composition, fiber alignment and mechanical stiffness. Importantly, these biophysical modifications influence melanoma cell, fibroblast and immune cell behaviors, generate intra-tumoral heterogeneity, promote metastasis and compromise anti-melanoma treatments. Furthermore, we give insight into how melanoma cells react to these architectural changes, as they interact directly with the ECM *via* transmembrane receptors, in a reciprocal manner, which influences melanoma cell migration, invasion and phenotypic tumor states. Notably, we provide evidence on how ECM composition, alignment and stiffness contribute to melanoma cells evading therapies and provide a perspective on current and future strategies to normalize the drug protective ECM in the TME.

## Author contributions

AP and ST-D contributed equally to the conceptualization, writing and editing of this review. All authors contributed to the article and approved the submitted version.

## Funding

AP is a postdoctoral research fellow whose fellowship is funded by the French National Institute for Cancer.

## Acknowledgments

The authors acknowledge grant support from the French League for Cancer (équipe labellisée 2022), the French National Institute for Cancer (INCA N°12673) and the French-South African PHC PROTEA project (N° 46471YC).

## Conflict of interest

The authors declare that the research was conducted in the absence of any commercial or financial relationships that could be construed as a potential conflict of interest.

## Publisher’s note

All claims expressed in this article are solely those of the authors and do not necessarily represent those of their affiliated organizations, or those of the publisher, the editors and the reviewers. Any product that may be evaluated in this article, or claim that may be made by its manufacturer, is not guaranteed or endorsed by the publisher.

## Glossary

**Table:**

ACD	Adrenocortical dysplasia protein homolog
AhR	Aryl hydrocarbon receptor
AJCC	American Joint Committee on Cancer
AKT	Protein kinase B
AP-1	Activator protein 1
AXL	AXL receptor tyrosine kinase
BAP1	ubiquitin carboxyl-terminal hydrolase BAP1
BRAF	B-Raf proto-oncogene
BRN2	POU domain
class 3	transcription factor 2
CAFs	Cancer associated fibroblasts
CCND1	Cyclin D1
CDK4	Cyclin-dependent kinase 4
iCAFs	Inflammatory cancer associated fibroblasts
myCAFs	Myofibroblast cancer associated fibroblasts
CD29	β_1_integrin
CDKN2A	Cyclin Dependent Kinase Inhibitor 2A
CTLA-4	Cytotoxic T-lymphocyte-associated protein 4
DDRs	Discoidin domain receptors
ECM	Extracellular matrix
EDA	Extra domain
EMILIN-2	Elastin microfibril interface-located protein 2
EMT	Epithelial to mesenchymal transition
ERK	Extracellular signal-regulated kinase
FAK	Focal adhesion kinases
FAPα	Fibroblast activation protein-α
FDA	Food and Drug Administration
GAS6	Gamma-carboxyglutamic acid (Gla)-containing protein
IKKα	IkB kinase α
LOX	Lysyl oxidase
MAFs	Melanoma associated fibroblasts
MAPK	Mitogen-activated protein kinase
MEK	Mitogen-activated protein kinase kinase
MC1R	Melanocortin 1 Receptor
miRNA	MicroRNA
MITF	Melanocyte inducing transcription factor
MMPs	Matrix metalloproteinases
MRD	Minimum residual disease
MRTF	Myocardin-related transcription factor A
NCSC	Neural crest stem cell
NF-1	Neurofibromin 1
NF-kB2	Nuclear factor kappa B subunit 2
NIK	NF-k-B-inducing kinase
NRAS	NRAS Proto-Oncogene GTPase
PAX3	Paired box gene 3
PDAC	Pancreatic ductal adenocarcinoma
PDGFRβ	Platelet derived growth factor receptor-β
PD-1	Programmed cell death protein 1
PD-L1	Programmed death-ligand 1
PDX	Patient derived xenograft
POT1	Protection of telomeres 1
PTEN	Phosphatase and tensin homolog
RGP	Radial Growth Phase αSMA α smooth muscle actin
SOX9/SOX10	SRY-Box Transcription Factor 9/10
SPARC	Secreted protein acidic and cysteine rich
S100A4	Fibroblast specific protein 1
TAZ	Transcriptional coactivator with PDZ-binding motif
TEAD	TEA domain transcription factor 1
TERF2IP	Telomeric repeat-binding factor 2-interacting protein 1
TERT	Telomerase reverse transcriptase
TNM	tumor
nodes	and metastases
TME	Tumor microenvironment
VGP	Vertical growth phase
YAP	Yes-associated protein
